# Comparison of Solidification Characteristics between Polymer-Cured and Bio-Cured Fly Ash in the Laboratory

**DOI:** 10.3390/polym15051107

**Published:** 2023-02-23

**Authors:** Yinggang Jia, Yuhan Liu, Jian Chen

**Affiliations:** 1 School of Engineering and Technology, China University of Geosciences, Beijing 100083, China; 2 Key Laboratory of Luminescence Analysis and Molecular Sensing (Southwest University), Ministry of Education, College of Chemistry and Chemical Engineering, Southwest University, No. 2 Tiansheng Road, Beibei, Chongqing 400715, China

**Keywords:** polyacrylamide, enzyme-induced carbonate precipitation, fly ash, unconfined compressive strength, wind erosion rate

## Abstract

Fly ash (FA) usually causes air and soil pollution due to wind erosion. However, most FA field surface stabilization technologies have long construction periods, poor curing effects, and secondary pollution. Therefore, there is an urgent need to develop an efficient and environmentally friendly curing technology. Polyacrylamide (PAM) is an environmental macromolecular chemical material for soil improvement, and Enzyme Induced Carbonate Precipitation (EICP) is a new friendly bio-reinforced soil technology. This study attempted to use chemical, biological, and chemical-biological composite treatment solutions to solidify FA, and the curing effect was evaluated by testing indicators, such as unconfined compressive strength (UCS), wind erosion rate (WER), and agglomerate particle size. The results showed that due to the viscosity increase in the treatment solution, with the increase in PAM concentration, the UCS of the cured samples increased first (from 41.3 kPa to 376.1 kPa) and then decreased slightly (from 376.1 kPa to 367.3 kPa), while the wind erosion rate of the cured samples decreased first (from 39.567 mg/(m^2^·min) to 3.014 mg/(m^2^·min)) and then increased slightly (from 3.014 mg/(m^2^·min) to 3.427 mg/(m^2^·min)). Scanning electron microscopy (SEM) indicated that the network structure formed by PAM between the FA particles improved the physical structure of the sample. On the other hand, PAM increased the nucleation sites for EICP. Due to the stable and dense spatial structure formed by the “bridging” effect of PAM and the cementation of CaCO_3_ crystals, the mechanical strength, wind erosion resistance, water stability, and frost resistance of the samples cured by PAM-EICP were increased significantly. The research will provide curing application experience and a theoretical basis for FA in wind erosion areas.

## 1. Introduction

Fly ash (FA) is a kind of powdered mineral residue produced by coal combustion in thermal power plants [1]. In recent years, with the continuous development of thermal power, the emission of FA has increased rapidly. About 600 million tons of FA are produced annually in the world [2], while only a quarter of them are utilized [3]. A large amount of FA occupies lots of land resources. Meanwhile, the dust from the ash yard has caused serious secondary pollution to the local ecological environment, a problem that has attracted widespread attention [4]. Therefore, it is necessary to explore a kind of surface solidification technology (i.e., forming a “crust” with mechanical strength on the surface of FA fields) to prevent the loose particles of FA, which contains a lot of heavy metals from being blown away by the wind, so as to achieve the purpose of stabilizing the ash fields.

There have been many dust control technologies used in ash fields, such as engineering, water sealing, chemical, and vegetation methods [5]. The spray water sealing method is easy to operate, but it needs to be carried out frequently, and the effect is unsatisfactory due to the high porosity and poor water retention of FA [6]. Mulch and plant vegetation that control dust in ash fields are thorough and environmentally friendly. However, the mulch thickness usually requires more than 30 cm for plant vegetation, which is costly. Traditional chemical curing materials include asphalt emulsions, cement mixtures, alum, water glass, etc., which can mitigate and control the dispersion of loose particles [7] and prevent wind erosion effectively, but many of them may cause secondary pollution to the environment [8]. As a new chemical curing material, organic polymers (such as carboxymethyl cellulose, polyacrylamide (PAM), xanthan gum, etc.) have high ductility [6], which can be used to bind non-cohesive and dispersed geotechnical materials.

PAM is a linear water-soluble polymer with a large number of amide groups that easily form hydrogen bonds, making it more water-soluble and giving it a higher chemical activity. Studies have shown that polymers such as PAM have been widely confirmed to improve the shear strength and wind erosion resistance of soils by increasing the cohesion between the soil particles [9] without any toxic effects [10]. Kim and Palomino [11] improved the engineering properties of soft clay by using PAM with specific molecular weight and specific ionic classes; Bishop and David [12] pointed out that the incorporation of PAM increased the deformability of clay; Bhardwaj et al. [13] investigated the effect of PAM on the permeability properties of soil samples with different particle gradation and mineral composition; Miao et al. [5] found that the surface strength and wind erosion resistance of the PAM-cured desert soils were significantly improved. It is a new attempt to solidify FA using PAM. However, the soil “crust” formed by the polymer may degrade in a harsh environment, which will result in a reduction in the strength of the cured sample [14]. Therefore, there is an urgent need to improve and optimize the curing effect of FA based on the chemical curing method.

Biological curing technology has been widely used to improve soil properties [15,16] because of its characteristics which include being economical, durable, stable, and eco-friendly [17]. Enzyme-Induced Carbonate Precipitation (EICP) [18] has been applied in soil solidification [19], and the cost is low due to urease being enriched in many plants, such as beans [20]. CaCO_3_ crystal is formed when Ca^2+^ is combined with CO_3_^2−^, while the CO_3_^2−^ is generated by the hydrolyzation of urea through urease in the process of EICP [21], and then the mineralized products cement loose particles together. Gao et al. [22] conducted a field curing test on sandy soils using soybean urease extract, and the results showed that the surface strength and the wind erosion depth of the sandy soils were treated with EICP for 30 days were about 306.2 kPa and 0 mm, respectively. However, the surface of the FA sample is smooth, and no organic matter is attached to it, which will lead to a lack of CaCO_3_ crystal nucleation sites in the process of EICP and result in a disordered aggregation of the generated CaCO_3_ and a formation of brittle damage in the end. To improve this kind of defect, it may be meaningful to add a polymer (e.g., PAM) to the EICP solution. 

This study is based on the surface activity of the polymer PAM and the cementation properties of the biological technology EICP to cure FA. Although most previous studies have focused on curing sand and soil using PAM and EICP, respectively, there was little research on improving the engineering characteristics of loose particles by combing PAM and EICP [5]. Moreover, it is a new attempt to cure FA by PAM and EICP. Therefore, this research aims to compare and evaluate the effect of PAM and EICP in curing FA by unconfined compressive strength (UCS), wind erosion rate (WER), and agglomerate particle size of the sample.

## 2. Materials and Methods

### 2.1. Materials

#### 2.1.1. FA

**Figure 1:**
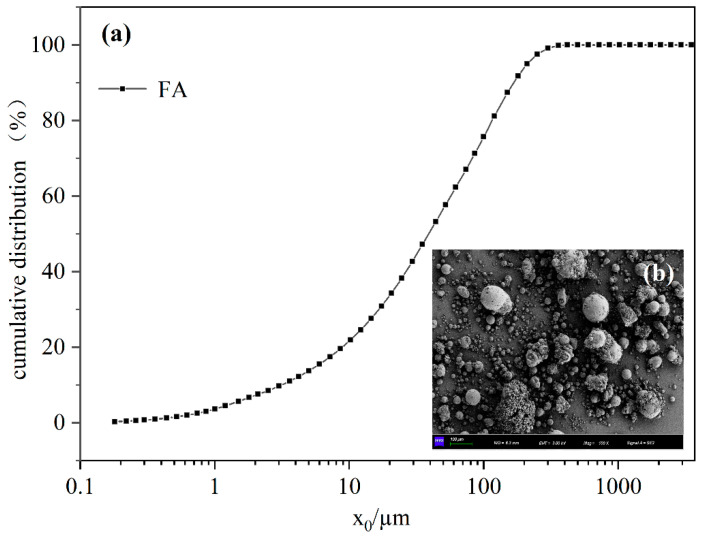
Particle size distribution curve (**a**) and SEM image (**b**) of FA.

The FA used for the study was taken from the coal power integration base in the Dananhu area of Hami, Xinjiang. The main chemical contents of the FA samples were SiO_2_ and Al_2_O_3_ (Table 1). The FA belongs to “Class F” (i.e., the content of CaO in the FA was less than 10% [4]), which was a low-calcium FA. The particle size of the sample was concentrated in the range of 0.005–0.2 mm (Figure 1), and the average particle size was 0.097 mm. SEM images indicated that the virgin FA was highly dispersive.

#### 2.1.2. Chemical Reagents

All of the chemical reagents were analytical grades. PAM was the anionic type with a relative molecular mass of 12 million, produced by Tianjin Zhiyuan Chemical Reagent Co. The PAM was dissolved in deionized water for 30 min at room temperature to form a solution with concentrations ranging from 0.2 g/L to 1 g/L. Anhydrous CaCl_2_ was selected as the exogenous calcium for the mineralization reaction due to it having the largest calcium carbonate production rate [23], which was a white powder with a relative molecular mass of 110.98; the urea was a white crystalline powder with a relative molecular mass of 60.06, both above chemicals were produced by Sinopharm Chemical Reagent Co. (Shanghai, China). Anhydrous CaCl_2_ and urea were dissolved with deionized water to a constant concentration of 0.3 mol/L and 1 mol/L, respectively.

#### 2.1.3. Urease

The urease was a kind of polysaccharide produced by Nanjing Biological Products Co., Ltd. (Nanjing, China). The urease activity (define: one unit causes the formation of two micromoles of ammonia per minute at pH 8.0 at 37 °C) was about 276 u/mg at 37 °C. In order to precisely control the urease activity in each sample during the test, a certain amount of urease powder was dissolved into a dilute solution of 13.8 u/mL with deionized water and then used immediately.

### 2.2. Sample Preparation

#### 2.2.1. Preparation

The virgin FA was passed through a 0.5 mm standard sieve to remove withered grass, various debris, and large FA particles which naturally agglomerated in the air. In order to meet different test requirements, the samples were divided into two groups (i.e., large samples and small samples). The large samples were mainly used for wind erosion tests, while the small samples were mainly used for mechanical tests. The large samples were made as follows: loading FA (about 100 g) above the top of the tray (the size of the tray was 17.5 cm × 11.3 cm × 2.2 cm (length × width × height) and then tapping along the circumference of the tray gently 10 times to make the samples dense. The small samples were made as follows: the sample cup (bottom diameter of the cup was 3.5 cm) was gently knocked 10 times after being filled with FA (about 100 g) to ensure the dry density of each sample was roughly the same.

#### 2.2.2. Solution

The treatment solution was divided into a blank control group (including the virgin FA sample and the sample treated with deionized water); a salt group; a PAM group; an EICP group; and a PAM-EICP group, which mainly explored the effect of the changes in concentration of PAM on the curing effect. The detailed composition of different treatment solutions is shown in Table 2.

For large samples, the treatment solution was sprayed evenly on the surface of the ashtray using a spray at a dosage of 0.45 L/m^2^; for small samples, every 10 g of FA was sprayed with 3 mL of treatment solution because of the liquid limit value of which was 32% [6]. All types of samples were provided with 3–12 groups of parallel samples (the number of parallel groups was adjusted according to the needs of the test). To prevent the impact of the water mist from damaging the surface of the sample, the vertical distance from the nozzle of the spray to the surface of the sample was 25 cm when sprayed the treatment solution. The spray was completed in a short time to minimize the biochemical reaction process in the sprayer.

#### 2.2.3. Maintenance

All treated samples (including the blank control group) were maintained at room temperature for 10 days (temperature: 28.4 °C; humidity: 49.4%) to provide sufficient time for the process of chemical and/or physical reactions between the treatment solution and FA, and then the samples were dried in an oven (temperature: 60 °C; time: 12 h) before the curing performance test.

### 2.3. Curing Performance Test

The following tests were carried out to research the curing properties of the prepared samples.

#### 2.3.1. UCS

The UCS has been used by many scholars to test the strength of bio-cement-reinforced soils, which was simple and rapid. The SLB-1 stress-strain controlled triaxial shear penetration tester (manufactured by Nanjing Soil Instrument Factory Co., Ltd., Nanjing, China) was used to conduct the UCS test. The measured indenter was flat, and the cross-sectional area was about 10 cm^2^. The sample was compressed at a uniform strain rate of 0.5 mm/min. The diameter of the sample for this experiment was 3.5 cm, and the height was about 8 cm. The detailed operation method was referred to as the Standard for Geotechnical Testing Method (GB/T 50123-2019).

#### 2.3.2. Screening Test

Mechanical oscillation and screening tests were carried out to evaluate the cementing effect of different treatment solutions on the FA particles. A GS-86 electric oscillating sieve machine (manufactured by Shangyu Sieve Factory, Shaoxing, China) was used as testing equipment, and the standard inspection sieves of 0.5 mm, 1.46 mm, and 2.5 mm were moved from bottom to top of it, the number of sieve vibrations was 1400 rpm and the oscillation time was continued for 15 min. The cured FA samples were placed on the 2.5 mm standard inspection sieve and weighed to determine the percentage content of the samples sieved by different mesh sieves.

#### 2.3.3. Wind Tunnel Test

The wind tunnel was used to test the windproof and ash-fixing performance of the cured samples. The samples were placed on the wind tunnel bottom frame, which was fixed on a slope of 10°. The total length of the wind tunnel machine was 60 cm, and the cross-section was 40 × 25 cm^2^. The wind speed was adjusted by an anemometer. The change of the sample mass (i.e., the amount of wind erosion) was weighed at a wind speed of 10 m/s (corresponding to wind class 5) and at a continual time of 18 min in the wind tunnel, the results of which were accurate to 0.001 mg. The wind erosion rate was calculated according to Equation (1),

*WER* = (*m*_0_ − *m*_1_)/(*s*·*t*) (1)

where *WER* was the wind erosion rate, mg/(m^2^·min); *m*_0_ was the mass of FA before wind erosion, mg; *m*_1_ was the mass of FA after wind erosion, mg; *s* was the surface area of the sample, m^2^; *t* was the wind erosion time, min.

#### 2.3.4. Water-Stability Test

The water stability of the surface for cured FA was an important reference index to evaluate the curing effect. The rainfall process was simulated by spraying deionized water above the samples. Every large sample was sprayed with about 10 g, while the small samples were sprayed with about 15 g of deionized water each time and then dried in an oven at 60 °C for 24 h. Thus the process was one wet-dry cycle, and so on for three cycles to determine the UCS and WER of these samples.

#### 2.3.5. Freeze-Thaw Test

The repeated seasonal freeze-thaw cycles might damage the structure of FA [24]. For this reason, the frost-resistance of cured samples was evaluated by freeze-thaw cycles tests: about 5 g of deionized water was sprayed for each large sample, and approximately 10 g of deionized water was sprayed for each small sample, and after that, these treated samples were put in the refrigerator at −20 °C for 12 h immediately and then dried at 60 °C for 12 h. This process was one freeze-thaw cycle, and so on three times to determine its UCS and WER.

#### 2.3.6. Particle Size Test

Particle size test referenced Particle Size Analysis-Laser Diffraction Methods (GB/T 19077-2016). The samples for the test met the requirements of the standard. The particle size of the FA in the liquid phase was tested by laser particle size analyzer HELOS (H2566) & QUIXEL, the dispersion medium was water, and the pump speed was 790 rpm. To obtain the particle size gradation, the test results were analyzed through the software PAQXOS (Sympatec, Clausthal-Zellerfeld, Germany).

#### 2.3.7. SEM

The SEM was performed to elucidate the morphological and structural differences of the crusts for cured FA samples. The instrument used for SEM was ZEISS Gemini 300 with Schottky field emission electron gun, the detectors of which were Inlens and ET secondary electron detectors, and the type of Energy Dispersive X-ray Spectroscopy (EDX) was Smartedx.

#### 2.3.8. X-ray Diffraction (XRD) Analysis

XRD analysis was used to characterize the mineral composition and identify the generated crystal types of the treated FA. The samples were scanned continuously using Smart lab (RIKEN, Tokyo, Japan) with a scanning speed of 10°/min, and the 2θ correction value was equal to 0.002°.

#### 2.3.9. Chemical Component Analysis

The chemical composition analysis of major elements of the FA samples was completed at the Analytical Laboratory Beijing Research Institute of Uranium Geology. The major elements were determined by XRF (Panaco Axios^MAX^) method, according to standard GB/T14506.28-2010, and the loss on ignition standard was GB/T14506.34-2019, with an analysis accuracy of 5%.

### 2.4. Statistical Analysis

The results of the UCS test, screening test, and wind tunnel test were performed by SPSS v.25.0 for Windows (IBM Inc., Chicago, IL, USA). The results of the UCS test, Screening test, Wind tunnel test and XRD test were plotted and compared using Origin 9.8 (Origin Lab Corp., Northampton, MA, USA).

## 3. Results and Discussion

### 3.1. Results of UCS

**Figure 2:**
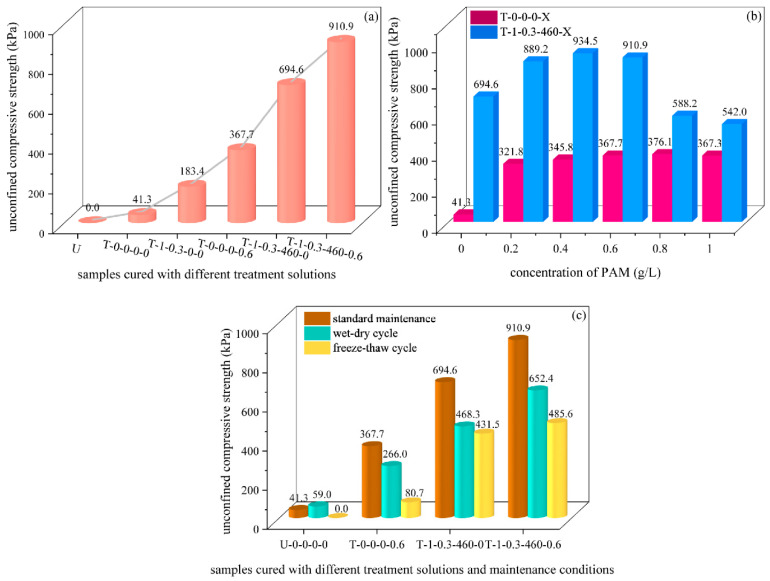
UCS of samples cured with different treatment solutions. (**a**) UCS of cured FA samples with different treatments (untreated (control), deionized water treatment, salt treatment, PAM treatment, EICP treatment, PAM-EICP combination treatment); (**b**) UCS of samples treated with different PAM concentrations; (**c**) UCS of samples after standard maintenance, wet-dry cycles, and freeze-thaw cycles.

Compared with the samples treated with deionized water, the UCS of the samples cured by salt (T-1-0.3-0-0), PAM (T-0-0-0-X), and PAM-EICP (T-1-0.3-460-X) were enhanced significantly (Figure 2a).

The relationship between the UCS of the sample and the concentration of PAM is shown in Figure 2b. The UCS of the sample treated with 0.2 g/L PAM reached 321.8 kPa, which was eight times higher than that of the blank control group (samples treated with deionized water only); the UCS of the sample increased by 7%, 6%, and 2%, respectively for each 0.2 g/L increase in the concentration of PAM solution, and the peak value was 376.1 kPa (T-0-0-0-0.8); along with the concentration of PAM solution increased continuously, the UCS of the sample decreased, which probably due to the low porosity of the FA affected the permeability of the high-viscosity solution which was caused by the high concentration of PAM [6].

The UCS of samples treated by EICP (i.e., urea concentration: 1 mol/L; CaCl_2_ concentration: 0.3 mol/L; urease activity: 460 u) was 17 times higher (694.6 kPa) than that of the blank control group, indicating the CaCO_3_ crystals generated by the mineralization reaction increased the bonding strength between FA particles (Figure 7b), this result was similar to that reported by Meng et al. [15]. Based on EICP concentration, the UCS of the samples increased significantly with the increase in PAM incorporation, and the maximum strength value (934.5 kPa) appeared when the PAM concentration increased to 0.4 g/L, which was nearly equal to the sum strength value (1040.4 kPa) of the sample treated with EICP (694.6 kPa) and PAM (345.8 kPa), respectively, indicating that the EICP and PAM have exerted their respective best performance in this compound ratio; the strength of the sample decreased slightly (910.9 kPa) when the concentration of PAM was 0.6 g/L. Along with the continuous increase in PAM concentration, the viscosity of the treatment solution increased, which resulted in the dispersibility of mineralization reactants (such as urease, urea, and CaCl_2_) in the samples, because of which, the uneven mineralization reaction might happen: more CaCO_3_ deposited at the top of the samples, and less CaCO_3_ deposited in the middle and bottom of the samples, this kind of uneven and incomplete structure reduced mechanical properties of the samples. Therefore, the UCS of the sample decreased dramatically. Previous studies have shown that when a lower concentration of polymer was used to cure loose particles, the microstructure of the polymer was grid-like [25], and the stability of the sample was enhanced. The failure interface was usually located inside the CaCO_3_ crystals for samples treated by biomineralization only [26], but the network structure formed by PAM provided an additional cementing effect [27], thus avoiding the fracture of the CaCO_3_ cementation part and improving the stability of FA samples.

The water-stability (after the wet-dry cycle) and freeze-resistance (after the freeze-thaw cycle) strength of the PAM-treated samples were reduced by 28% and 78% compared with the standard maintenance samples (Figure 2c), the result of which might be due to the dehydration of the PAM gel in the drying cycle, but the water remained available partially for the next wetting cycle because of its strong affinity for water [28]. At the same time, the degradation of the hydrogen bonds formed in the pores between the FA particles was accelerated during repeated freeze-thaw cycles, which led to a reduction in the strength and stiffness of the PAM-treated samples because of the breakage of the structural bonds. Compared with the standard maintenance samples, the water-stability and freeze-resistance strength of the EICP-treated samples were reduced by about 33% and 38%, respectively, indicating that the water-stability of the EICP-treated sample was slightly worse than that of the PAM-treated sample, the sandy soils treated by similar methods showed a similar result [28]. When the EICP was added to the PAM solution with a concentration of 0.6 g/L, the water-stability and freeze-resistance strength of the sample increased by 145% and 502% compared with the PAM-treated sample.

### 3.2. Analysis of Agglomerated Particles

**Figure 3:**
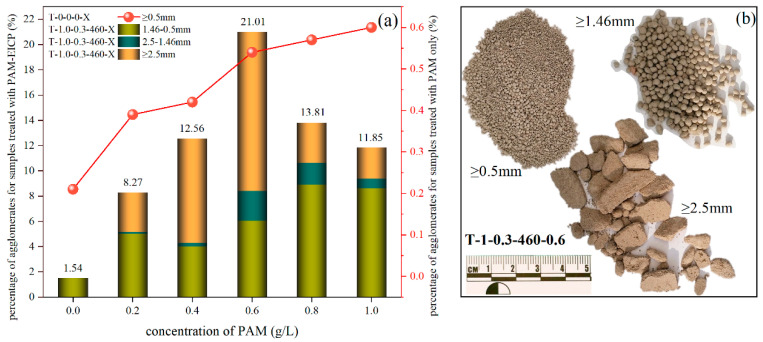
Percentage of agglomerates (i.e., the particle size of the FA was equal to or more than 0.5 mm) in the total mass of the samples cured with different treatment solutions. (**a**) with the increase in the PAM concentration, the percentage of agglomerated particles (≥0.5 mm) showed an increasing trend, dot-line diagram for the samples treated by PAM, column graph for the samples treated by PAM-EICP; (**b**) particle size of sample T-1-0.3-460-0.6 after sieving.

The concentration of PAM had an important effect on the particle size distribution of the agglomerates formed by the solidification of FA (Figure 3a dot-line diagram). With an increase in PAM concentration, the percentage of agglomerated particles (≥0.5 mm) showed an increasing trend. When the concentration of PAM increased from 0 to 0.2 g/L, the agglomerates larger than 0.5 mm of the samples increased rapidly, which reflected that the addition of PAM contributed to the rapid increase in the cement strength of FA. When the PAM concentration was increased to 0.4 g/L, there was a further increase in agglomerates, but the contribution to the cementation strength of FA was limited because the PAM concentration was still low. Therefore, the trend line change from 0.2 to 0.4 (dot-line, FA samples cured by PAM) rose relatively slowly. Moreover, the upward trend of the trend line became slower when the concentration of PAM was greater than 0.6 g/L. In addition, the particle size and percentage of agglomerates increased significantly with the addition of EICP in the PAM solution, and the maximum particle size was up to 40 mm (Figure 3b), the maximum mass proportion of the agglomerated particles in the sample was 21.01% (Figure 3a column graph), indicating that the incorporation of EICP in the PAM treatment solution could effectively promote the formation of large agglomerates of FA particles, improve the structure of FA surface and raise the dust initiation wind speed (i.e., the frequency of possible dust lifting was reduced).

### 3.3. Analysis of Wind Erosion Resistance

**Figure 4:**
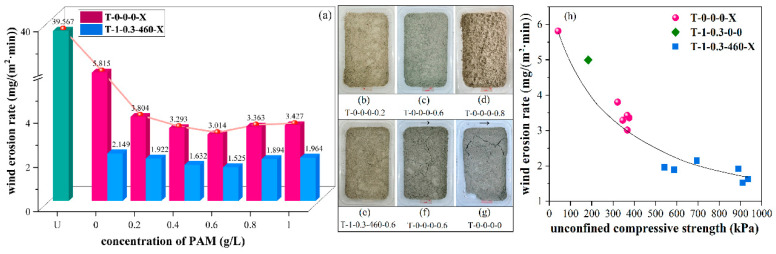
Wind erosion of samples treated with different solutions (the direction of the arrow in the physical photo represented the wind direction). (**a**) the WER of samples changed with the increase in PAM concentration; (**b**–**d**) the surface “crust” of the samples changed from smooth to rough after being cured with different concentrations of PAM; (**e**) the surface “crust” of the sample treated by PAM-EICP solution is denser; (**f**) FA particles could escape from cracks on the surface of the samples treated by PAM after wet-dry and freeze-thaw cycles; (**g**) the frost-resistance of the samples treated by deionized water was weak; (**h**) the correlation between the WER and the UCS.

The amount of wind erosion is one of the key indicators to quantify the curing effect. With the increase in PAM concentration, the WER of samples showed a decreasing trend (Figure 4a), which was because PAM bound the fine FA particles into large agglomerates. On the other hand, PAM penetrated the pores of FA particles and reduced the energy transfer between particles, which further improved the stability of FA particles. When the concentration of PAM increased to 0.8 g/L, the WER increased slightly. When comparing the surface morphology of the FA after being cured with different concentrations of PAM, it could be found that the surface “crust” of the samples changed from smooth to rough (Figure 4b–d) when the concentration of PAM increased continuously, which was mainly caused by inhomogeneous spray when the high viscosity solution was used. In the wind tunnel test, the airflow with fine FA particles was blocked by the raised obstacles on the surface of the sample and then flowed to both sides of them. As a result, the flow velocity increased on both sides of the obstacle, which quickly caused erosion to the leading edge and the side of the obstacle. It made lots of new erosion grooves on the surface of the sample by the new FA particles stripped from the obstacle. In addition, the airflow would detach and return to these grooves and hit and deepen them. Therefore, the flatness and solidity of the consolidation surface have an important influence on its resistance to wind erosion [29].

The mass loss of the samples was reduced by adding equal concentrations of EICP solution in different concentrations of PAM treatment solution, and the minimum WER was 1.525 mg/(m^2^·min), which was smaller than that of the samples treated with PAM solution (3.014 mg/(m^2^·min)) with the concentration of 0.6 g/L and EICP (2.149 mg/(m^2^·min)), respectively. In general, the consolidation strength of EICP was better than that of PAM, but the thickness of the consolidated layer was small [27]. The addition of PAM could further improve the performance of the samples by forming a denser and stronger “crust” (Figure 4e).

FA particles would escape from cracks on the surface of the samples (Figure 4f), which were formed by wet-dry and freeze-thaw cycles. However, the CaCO_3_ crystals generated in EICP acted as a connection point between particles and provided a binding force for the FA to resist freeze-thaw cycles [30]. Additionally, the spatial network structure formed by PAM also increased the stability of the sample. However, the frost resistance of the samples treated by deionized water was weak (Figure 4g), the samples showed failure points at the wind speed of 10 m/s, and along with the erosion of wind and the abrasion of FA particles, the failure points expanded into cracks rapidly with a mass loss of 10.552 mg/(m^2^·min), and the mass loss would be more serious if the wind speed continued to increase, similar results were carried by Naeimi and Chu [31].

There was a significant negative correlation between the WER and the UCS (Figure 4h). That was, as the UCS increased, the WER of the samples decreased (i.e., the wind erosion resistance increased), indicating that with the optimization of the curing scheme, the structural stability and strength of FA increased, and the windproof and ash-fixing effect was enhanced significantly.

### 3.4. Microscopic Analysis

**Figure 5:**
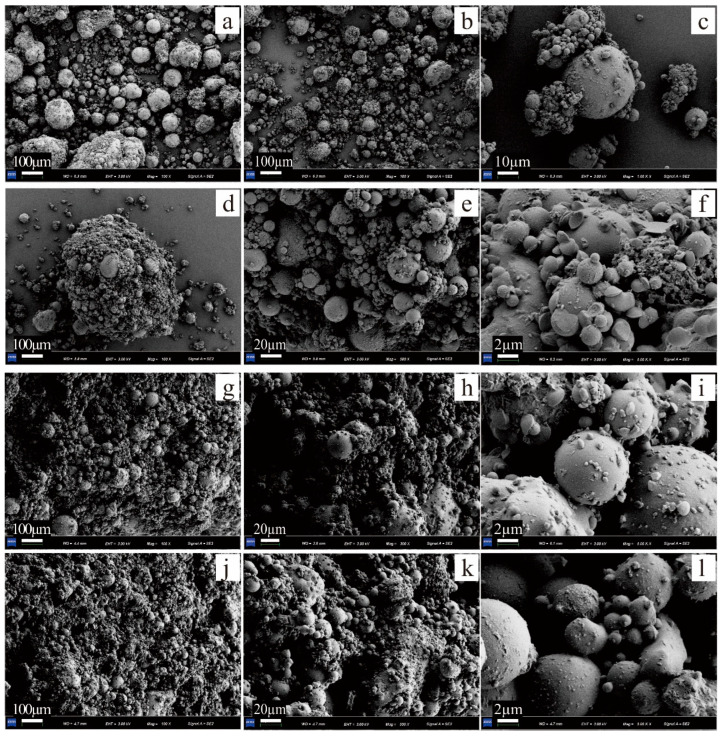
SEM images of FA cured with different treatment solutions. (**a**,**b**) the looseness of the samples treated by PAM and EICP; (**c**) the contact relationship between FA particles of the samples treated by EICP; (**d**–**f**): T-1-0.3-460-0.4; (**g**–**i**): T-1-0.3-460-0.6; (**j**–**l**): T-1-0.3-460-0.8.

The standard maintenance samples were dried, and their surface “crust” was taken for SEM. SEM images showed that the size of the agglomerates formed by the PAM and EICP were comparable (Figure 5a,b). The physical or chemical bonding between the PAM and FA particles resulted in the structure of the agglomerate being compacted. A similar structure was formed when acrylamide was used to control the dust [32]. There were many CaCO_3_ “bridges” (Figure 5c) generated by the mineralization reaction between the EICP-treated FA particles. When PAM and EICP solution were mixed, the agglomerate particles increased dramatically (Figure 5d); the degree of compactness (Figure 5e,h,k) and structural integrity (Figure 5g,j) of the samples increased significantly with the increase in PAM concentration, and the porosity decreased first and then increased slightly (Figure 5f,i,l). This also explained why the UCS and wind erosion resistance of FA samples treated with PAM-EICP composite solution were higher than those cured by a single chemical or biological solution: the PAM solution could flow, adhere, and wrap between particles in FA due to its viscosity and fluidity. The contact area between FA particles increased during the flow of colloids formed by PAM, which also provided more nucleation sites for CaCO_3_ and made up for the defect of little nucleation sites in EICP technology. When the residual water of the colloid was dehydrated and hardened, the interaction between FA particles wrapped by colloid and CaCO_3_ crystals was tighter, which was reflected in improving the UCS and wind erosion resistance of FA in macro characteristics.

On the basis of equal concentration of EICP solution, when the incorporation of PAM concentration was 0.8 g/L, the surface of FA particle was mostly wrapped by a large number of PAM colloids, and there were many CaCO_3_ particles with smaller particle sizes distributed on the surface of the colloids (Figure 5l). It has been shown that the higher the viscosity of the solution, the smaller the particle size of the CaCO_3_ crystals [33].

**Figure 6:**
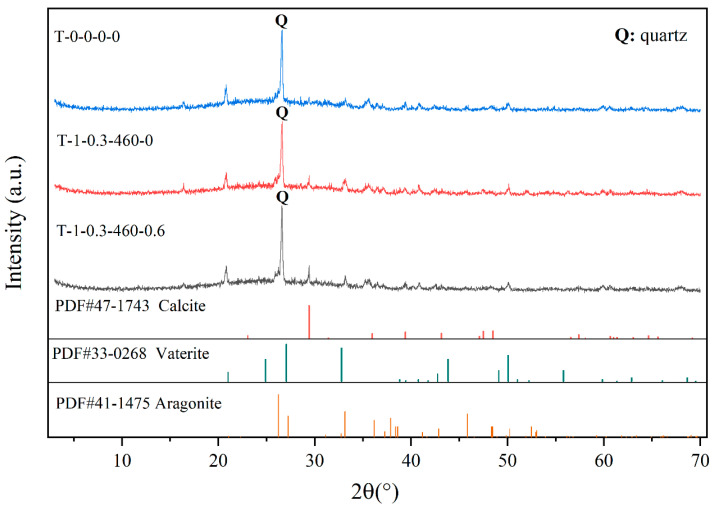
XRD patterns of the “crust” for samples cured by different methods. The red lines, green lines, and tawny lines at the bottom of the Figure are the PDF references for calcite (#47-1743), vaterite (#33-0268), and aragonite (#41-1475), respectively.

XRD patterns are often used as a tool to distinguish mineral types [34]. The peaks and intensities of crystals of the anhydrous forms for CaCO_3_, namely vaterite (hexagonal), aragonite (orthogonal), and calcite (rhombohedral), were different in XRD patterns [35]. The results showed that calcite appeared in the samples treated by EICP and PAM-EICP compared with the control group, and the peak intensity of calcite formed in the samples treated by PAM-EICP was higher than that of the samples treated by EICP only (Figure 6).

### 3.5. Analysis of Curing Mechanism

**Figure 7:**
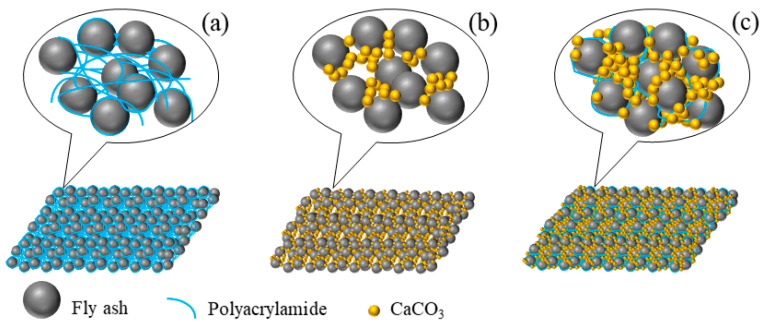
Schematic diagrams for curing mechanisms of FA treated with chemical (**a**), biological (**b**), and chemically-biological (**c**) methods.

PAM is a long-chain molecule with a large number of active polar groups (such as amide group (-CONH_2_)) on the side chain, which could form a liquid bridge between particles by adsorption on the surface of FA through van der Waals force, electrostatic attraction and hydrogen bonding (Figure 7a). PAM molecules could adsorb multiple FA particles at the same time and link them together to form larger agglomerates, thus strengthening the integrity and stability of FA. It has been found that the polymer tended to aggregate at the contact position of sand particles [36]. The long chain of PAM reduced the energy transfer between FA particles and increased the smoothness and toughness of the sample surface [6], thus decreasing the vibration of FA when high-speed airflow passed.

The pH and CO_3_^2−^ concentration increased in the solution due to the hydrolyzation of urea by urease when added the EICP was in the PAM solution. At the same time, the negatively charged groups formed by PAM hydrolysis also had high cohesion energy for opposite charges (such as Ca^2+^) and provided binding sites for Ca^2+^ [37] to make the Ca^2+^ concentration locally supersaturated and accelerate the nucleation process of CaCO_3_. After that, the long PAM chains not only selectively adsorbed on the nano-CaCO_3_ crystal surface but also intertwined and wrapped on the FA particles, forming a relatively stable and densely filled spatial skeleton mesh structure (Figure 7c), which played a role of flexible filling and reinforcement between the pores of FA particles and improved the physical structure of the FA body to reduce the generation of microcracks.

## 4. Conclusions

The paper compared the curing characteristics of the FA samples treated with chemical, biological, and chemical-biological composites, respectively, and obtained the following conclusions:

(1) Compared with the deionized-treated sample, the curing effect of the sample treated by salt, PAM, EICP, and PAM-EICP composite solution was enhanced sequentially.
(2) The spatial network structure formed by PAM improved the mechanics, wind erosion resistance, and water stability of the FA samples. When the concentration of PAM ranged from 0.6 g/L to 0.8 g/L, the UCS and wind erosion resistance of the sample appeared to be the optimal value. However, it reduced when the concentration of PAM was increased continuously.
(3) The CaCO_3_ crystals enhanced the cohesion of the sample by cementing FA particles in the EICP process, which also showed good freeze-thaw stability.
(4) The addition of PAM provided more nucleation sites for the formation of CaCO_3_ in the EICP process and increased the particle size of FA agglomerates. SEM revealed the stable and dense spatial structure formed by the cementing action of PAM and mineral crystals. At the same time, the uneven mineralization reaction and lots of small crystals were generated when the concentration of the PAM solution was too high.

More than half of the world’s FA is piled up in situ. High winds can easily strip FA from the surface of ash dumps. FA particles suspended in the air contain many heavy metal elements, causing serious impacts on the local ecological environment and human health. Therefore, the problem of surface stability of FA dumps needs to be solved urgently. The paper comprehensively evaluated the curing effect of each material through the indicators of wind erosion rate and strength. In addition, the stability of the curing performance of different materials under extremely harsh conditions was investigated in detail by modeling the wet-dry and freeze-thaw cycles in natural conditions. The experimental results provide a possibility for the practical application of polymer and EICP technologies to control wind erosion in FA fields. 

However, there are also many challenges. For example, the viscosity is significantly enhanced when the PAM (especially high-dose PAM) is configured into an aqueous solution, which affects the crusting effect. Further optimization should be explored in the application method of PAM.

## Figures and Tables

**Table 1:** Major element (wt %) compositions of FA.

SiO_2_	Al_2_O_3_	Fe_2_O_3_	MgO	CaO	Na_2_O	K_2_O	MnO	TiO_2_	P_2_O_5_	Loss on Ignition	FeO
56.32	21.67	4.92	2.15	8.97	1.41	1.64	0.053	1.02	0.09	1.73	1.95

**Table 2:** Composition of different treatment solutions.

No.	Composition	Urea (mol/L)	CaCl_2_ (mol/L)	Urease (Activity: u)	PAM (g/L)
1	U	0	0	0	0
2	T-0-0-0-0	treated with deionized water			
3	T-1-0.3-0-0	1	0.3	0	0
4	T-0-0-0-0.2	0	0	0	0.2
5	T-0-0-0-0.4	0	0	0	0.4
6	T-0-0-0-0.6	0	0	0	0.6
7	T-0-0-0-0.8	0	0	0	0.8
8	T-0-0-0-1	0	0	0	1.0
9	T-1-0.3-460-0	1	0.3	460	0
10	T-1-0.3-460-0.2	1	0.3	460	0.2
11	T-1-0.3-460-0.4	1	0.3	460	0.4
12	T-1-0.3-460-0.6	1	0.3	460	0.6
13	T-1-0.3-460-0.8	1	0.3	460	0.8
14	T-1-0.3-460-1	1	0.3	460	1.0

Note: in the columns of “composition” of the table, the letter “U” indicates the untreated sample; “T” represents the treated sample, and the number means the amount of each chemical reagent.

## Data Availability

The data presented in this study are available on request from the first author and corresponding author.
